# Good Breeding

**Published:** 1875-09

**Authors:** 


### GOOD BREEDING.

During a recent tour through the sections of country resorted to in summer for health and recreation, we were struck with the very general evidences of ill-breeding which were visible almost everywhere. We were travelling solitary and alone, and therefore had abundant opportunity to observe and reflect.

It has been remarked that in no position in life are the evidences of good or bad "bringing-up" so apparent as at the table. At the table, in the act of partaking of food, men are either gentlemen or boors.

Nothing is easier than the acquisition of good table manners, and perhaps nothing is more rare. Of course manners of any sort acquired late in life do not sit gracefully upon the wearer. To come naturally, and as a matter of course, good manners should be ingrained, as it were, inwrought, making a part of the daily life from childhood up. It is early association which, in nearly all cases, gives character to the subsequent life. If the father and mother eat like animals and talk like barbarians, the children cannot wholly escape contamination. A few generations of such people, unelevated by any refining intermixture, is sure to secure great degredation; and depravity is only a step lower down. Families and communities never remain long stationary: the tendency is either up or down. With Americans, the tendency is usually upward. Some son or daughter goes out from the rural home, knowing little of the unselfish amenities of refined and cultured life, but quick to see and apt to learn—and returns to infuse a new air over the rude circle. If he or she is wise and good, only excellent and valuable acquisitions are added to the family store; if weak and foolish, the worst attributes of the worst classes with which they have associated are liable to adhere to them. It is wonderful to notice how well-nigh universal all over the country, is the use of the knife for conveying food to the mouth. The object of the knife is chiefly to cut. It has other uses; as, for example, to spread butter; but it never was designed to be thrust into the mouth. It is a very awkward instrument for this purpose, because its shape makes it necessary that the elbow should be elevated and protruded to keep the implement on a level. This use of the knife is not tolerated among cultivated people, and is especially to be deprecated at tables not supplied with a butter-knife, as is often the case in the country hotels. To take the knife out of the mouth and dip it into the common butter or salt-dish, is not by any means an uncommon occurrence, although a very disgusting one to the cleanly looker-on. The hotel-keeper who doesn't comprehend the ordinary decencies of life sufficiently to provide butter-knives and salt-spoons, and sugar-spoons, ought to abandon the traffic which he disgraces.

The noises made by the mouths of eaters are marvellous and disheartening. The supreme height over the lower animals to which cultivated man attains, as indicated in noiseless mastication, is fallen far short of by the average hotel denizen in the country.

The habit of reaching over the plate and immediate surroundings of a neighbor, is constant. It seems to be deemed improper to ask a fellow-guest to pass an article within his reach, but entirely decent to reach across his plate and seize it. This is a gross error. It is always polite to decorously ask for what is beyond our reach; it is always vastly ill-bred to pass hand and arm in front of our neighbor in order to secure it.

Nearly all the country hotels now-a-days place glasses full of wooden tooth-picks on the table. These are eagerly seized upon by some and energetically used during any periods of waiting. The effect upon the appetite of sensitive people may be left to the imagination. The fact that some things are to be done in private, and that cleaning the nails, and picking the teeth are of this sort, seems to be widely forgotton.

Good manners should be taught at home; but in thousands of homes there is nobody to teach them. The duty then devolves upon the school-teacher, who ought to be schooled up to the decent and unselfish and respectful amenities of cultivated society. In many cases he is not so schooled, and his example is nearly as harmful as that of the home circle. There seems, then, but one way to secure a correct demeanor, and that is by making it a branch of education. Let us have teachers of manners as well as of mental philosophy. Give us more etiquette and less Greek. Let us be decent if we cannot be deep. Let us be strong in good manners even though weak in book-lore. We will not admit that the engrafting of appropriate discipline in manners and politeness, would in any sense lessen the attention to other topics of education. The competent teacher will teach manners not by setting apart hours to consider behavior, but by a method less palpable. He will, first of all, set the example of goodness. We say goodness, for we are sure that ill-manners always imply something bad. The great companion-trait is selfishness, forgetfulness of the rights of others, of the presence of others, and of the respect which is due to the feelings of all human beings. The genuine teacher, then, will show in his own life, how sincerely and generously he respects the feelings of others, the small not less than the great. This example will surely be imitated by the best pupils. Vicious habits—and few habits are more vicious than those which render unhappy the human being with whom we are thrown in chance contact—should be kindly met with a private explanation of their impropriety. If a pupil is seen to scrape his finger nails publicly, he should be told that this act cannot be tolerated in public. If he uses a tooth-pick or carries it in his mouth, he should be assured that well-bred people never do these unpleasant things in the presence of others. If he is seen to spit on the floor, or in any public manner, he should be made to understand that he has done a rude and disgusting thing. If he blows his nose with his fingers, and without the interposition of a handkerchief, he should know that he has committed an unpardonable breach of decorum. If he lolls and lounges when he should sit or stand; if he carries his hands in his pockets; if he blows his food, instead of waiting for it to cool; if he conveys from his mouth by the aid of spoon or fork or knife, such portions of the food as are unfit to swallow, instead of quietly removing them with his fingers; if he has dirty hands when unemployed, if he hawks and spits and blows his nose when sitting at meals—for we have seen even this disgusting barbarism practiced of late;—if he spits at all except very privately; if he tucks his napkin under his chin, instead of holding it in his lap or using it to wipe his mouth or fingers; if he makes himself a nuisance by using tobacco or any other exceedingly dirty substance; if he sits at table with an accumulation of dirt beneath his finger-nails; if he neglects to keep his teeth clean; if, in short, he does anything indecent, uncivil, unclean or impolite, let the error be privately pointed out as often as it occurs, and the result will be, or ought to be, the eradication of the vice. With the young properly instructed in the decencies of refined and cultivated life, we may hope that the American people will one day become as famous for good-breeding and politeness as they are for energy and business capacity.

---

Far sweeter music to a true woman than the tone of a harp or piano touched by her hand, are the cheerful voices of husband and children, made joyous by her presence.

